# The functional role of time compression

**DOI:** 10.1038/srep25843

**Published:** 2016-05-16

**Authors:** Eckart Zimmermann, Christina Derichs, Gereon R. Fink

**Affiliations:** 1 Cognitive Neuroscience, Institute of Neuroscience and Medicine (INM-3), Research Centre Jülich, Germany; 2 Department of Neurology, University Hospital Cologne, Germany

## Abstract

Multisensory integration provides continuous and stable perception from separate sensory inputs. Here, we investigated the functional role of temporal binding between the visual and the tactile senses. To this end we used the paradigm of compression that induces shifts in time when probe stimuli are degraded, e.g., by a visual mask (Zimmermann *et al*. 2014). Subjects had to estimate the duration of temporal intervals of 500 ms defined by a tactile and a visual, masked stimulus. We observed a strong (~100 ms) underestimation of the temporal interval when the stimuli from both senses appeared to occur at the same position in space. In contrast, when the positions of the visual and tactile stimuli were spatially separate, interval perception was almost veridical. Temporal compression furthermore depended on the correspondence of probe features and was absent when the orientation of the tactile and visual probes was incongruent. An additional experiment revealed that temporal compression also occurs when objects were presented outside the attentional focus. In conclusion, these data support a role for spatiotemporal binding in temporal compression, which is at least in part selective for object features.

The high accuracy of temporal event perception by the human visual system is illustrated by, e.g., ball sport activities like tennis or baseball. Other obvious examples can be derived from the animal kingdom where an accurate estimate of the predator’s arrival may be decisive about survival. Despite the ecological relevance to correctly judge temporal durations, several illusions demonstrate a surprisingly strong susceptibility to interference resulting in altered time perception: Temporal intervals appear compressed during voluntary actions^1^, eye movements^2^, and shifts of attention^3^,^4^,^5^. In contrast, duration dilation occurs if i) an eye movement is directed towards an interval as in the stopped clock illusion^6^, ii) an ‘oddball’ is shown in a train of identical stimuli^7^, or iii) stimuli are made less predictable^8^. At least at first glance, these findings seem at odds with the necessity to accurately perceive time. Here, we explore the plasticity of time perception.

Many phenomena of temporal plasticity can be explained by the amount of attentional resources allocated to a time interval^9^ or the temporal order of events^10^. On the one hand, it has been shown that dividing attentional resources between different properties of the same stimulus can induce an underestimation of its duration^3^. For example, Cicchini and Morrone^5^ demonstrated that shifting attention to a distractor task leads to a compression of a temporal interval. On the other hand, stimuli with an abrupt onset, which capture attention transiently, are estimated to last longer^11^. An alternative account for this temporal plasticity has been offered by Pariyadath and Eagleman^8^ who suggested that predictability of the stimulus is responsible for its apparent duration.

How does plasticity of time perception fit with the ecological demand of accurate time perception in order to ensure survival? Is this plasticity only the by-product of a reduction of processing resources or does it subserve the need to classify stimuli in time? The so-called object correspondence problem is a constant challenge for the visual system, whenever the eyes move or blink or whenever a distracting flash of light or a mask interrupt the visual input. Plasticity of time perception and in particular time compression might be an important prerequisite for solving the correspondence problem. Matching stimulus correspondence becomes particularly pressing in a cross-modal situation. In order to provide coherence across multisensory stimulation, the brain needs to actively organize the perceptual experiences^12^. For example, multisensory binding has been investigated with temporal order judgements in a visual-tactile version of the ventriloquism effect^13^. The precision of these judgments was more accurate when the visual and tactile stimuli were presented in different spatial locations rather than in the same location, suggesting multisensory integration into a unitary percept^14^.

We have recently demonstrated that apparent time and space are compressed between two consecutively flashed objects if one of the stimuli is degraded by a briefly presented visual whole field mask^15^. Compression was strongest when the mask was shown in temporal vicinity of the stimulus, and disappeared when the mask occurred long before or long after the stimulus. We assumed that since masking leads to a generally impoverished probe signal, its spatial location becomes harder to detect. The poorer spatial representation makes the probe susceptible to attraction by a reference object with a strong spatial representation. We have also shown that compression in time and space is much stronger when the stimulus features of the presented objects are identical^15^. We therefore suggested that compression might be the result of a spatio-temporal binding process which matches and groups corresponding objects in case their spatial representation is weak.

In our current study, we investigated whether temporal compression also occurs for intervals defined by multisensory stimuli. We presented a tactile stimulus to the unseen hand and a masked visual stimulus in the same or a different position shortly thereafter. Subjects had to report the apparent temporal interval between the two stimuli. We found that the temporal interval was compressed when tactile and visual stimulation coincided spatially. The effect disappeared as a function of spatial distance between tactile and visual stimulus. The effect also depended on the correspondence between the stimuli, i.e., the effect disappeared when the tactile stimulus did not match the visual stimulus.

## Results

### Experiment 1: Visual-tactile temporal compression

**Figure 1:**
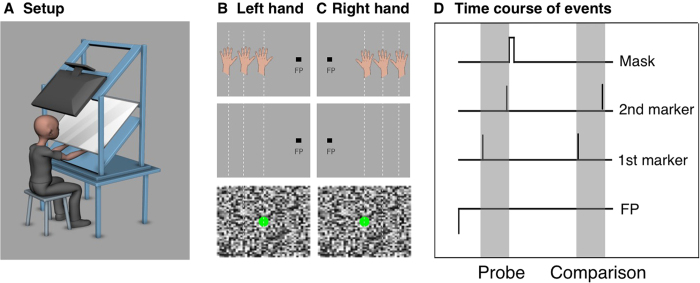
(**A**) Experimental set-up. The monitor was mounted upside down on a plexiglas construction. The image was reflected in a mirror below where it could be seen by the subject. The subject´s hand was placed on a further plane below the mirror. Here the tactile stimulation was applied. This construction enabled us to present visual stimuli at the monitor (reflected in the mirror) and tactile stimuli to the hand at similar perceived positions. Copyright 2016 Martin Hebestreit. (**B,C**) Possible positions of visual and tactile stimuli in the temporal compression experiment for tactile stimuli presented at the left and the right hand. The visual stimulus (green dot) was always presented at the screen center whereas the position of the tactile stimulus varied (indicated by the positions of the hand). It was presented either at the same position as the visual stimulus or displaced by 12° or by 24°. Fixation (black square) could be at the right or at the left side of the screen. Copyright 2016 Martin Hebestreit. (**D**) Timecourse of events for the temporal compression experiment. Each trial started with a fixation period of 1000 ms. We next presented the probe interval (shown by gray shaded area). The probe interval lasted 500 ms and started with a tactile stimulus applied for 50 ms. The probe interval ended with a visual stimulus flashed for 16 ms. This visual stimulus was covered by a mask of 50 ms duration. After a break of 1000 ms, the comparison interval (shown by gray shaded area) was presented. The comparison interval had a random duration of 200–800 ms. This interval was also started by a tactile stimulus presented for 50 ms and ended by a visual stimulus flashed for 16 ms.

We first sought to investigate whether binding of stimuli across the visual and the tactile senses might produce temporal compression. We asked subjects to judge the duration of a 500 ms interval, defined by a tactile impulse acting as the marker of its beginning and a visual impulse acting as the marker of its end (see Fig. 1). The visual impulse was followed by a whole-field random dot mask. Subjects had to compare the first against a second interval presented 1000 ms later, similarly defined by a tactile and a visual impulse. After the second interval, no mask was presented. The second interval was of variable duration and subjects were asked to indicate whether the first or the second interval appeared shorter. In order to test the role of object congruency, we varied the spatial distance between the tactile and visual interval markers. If the spatial congruency of objects is a cue for temporal binding then compression magnitude should increase as a function of how close objects are in space. In separate sessions we also tested to what extent multisensory temporal compression resembled within-sensory compression. To this end we used an identical set-up, but replaced the tactile by a visual interval start marker.

**Figure 2:**
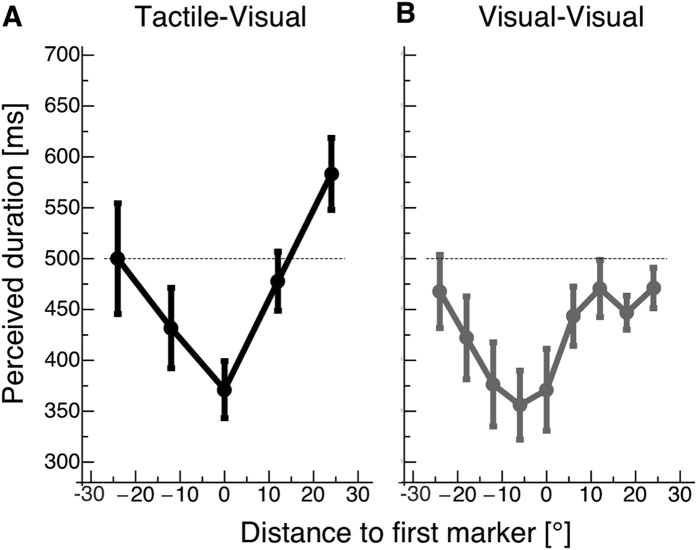
(**A**) Results of the visual-tactile time compression experiment. The perceived duration of the first interval is plotted for the three distances between tactile anchors and visual probes. The dotted line marks the physical duration of the first interval of 500 ms. Error Bars represent SEM. (**B**) Results of the visual-visual time compression experiment. Error Bars represent SEM.

Figure 2A shows the perceived duration of the first interval as a function of the distance between the visual stimulus and the tactile anchor. Tactile impulses were delivered to the left or right index finger in separate sessions. Since there were no significant differences between the left and right hands, we subsequently pooled the data. In Fig. 2A, the dotted line marks the physical duration of the probe interval of 500 ms. Data are shown for both fixation at the right and left side of the screen. When the stimulated finger was placed under the right part of the mirror (0 to 24°) the fixation point was projected onto the left part of the mirror. Likewise, if the stimulated finger was placed under the left part of the mirror (0 to −24°) the fixation point was projected onto the right part of the mirror. Since the finger position at 0° was tested for left and right fixation, data were pooled for this finger position. We found a strong reduction of the perceived duration of the probe interval (in relation to its physical duration of 500 ms) when visual and tactile stimuli were presented at corresponding locations (distance 0° in Fig. 2A): the temporal interval was underestimated by about 100 ms, which amounts to an underestimation of the interval’s total duration of 26%. With an increasing distance between tactile and visual stimulus, compression decreased and subjects estimated the interval duration almost veridical: When both stimuli were presented with a spatial separation of 12°, an underestimation of the temporal interval was still present but reduced while at a spatial separation of 24°, estimations of the interval duration were almost veridical.

Temporal estimations for purely visually defined intervals are shown in Fig. 2B. A very similar dependency of time compression on the spatial congruency of the interval markers was found. Again, when interval markers were closest in space, time compression was strongest, amounting up to 29% of the interval’s duration.

We calculated a two-way ANOVA of the perceived duration of the first interval with the factors “modality” (visual/tactile) and “position” (−24, −12, 0, 12, 24 deg). Please note that we used those visual-visual data bins for statistics whose positions corresponded to the visual-tactile data bins. We found a significant factor “position” (F(1,24) = 6.63, p < 0.001). No significant difference in the factor “modality” (F(1,6) = 0.80, p < 0.37) was found.

### Experiment 2: Correspondence matching

**Figure 3:**
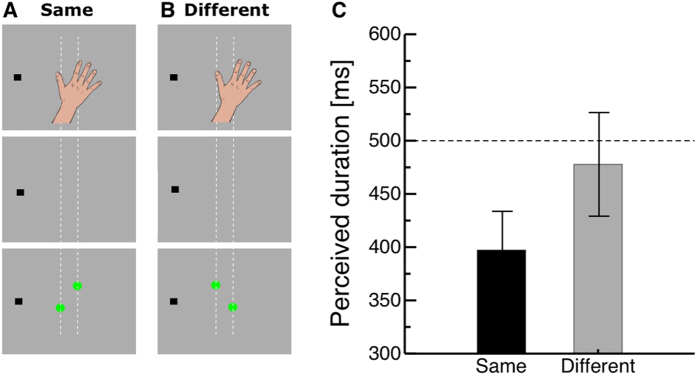
(**A,B**) Arrangement of visual and tactile stimuli in the correspondence matching experiment. In the same condition (**A**), tactile and visual stimuli are presented at corresponding positions. Copyright 2016 Martin Hebestreit. However, in the different condition (**B**) tactile and visual stimuli are presented in an opposite orientation. (**C**) Results of the correspondence matching experiment. The perceived duration of the first interval is plotted on the ordinate. The two arrangements of visual and tactile stimuli (same and different) are shown on the abscissa. The dotted line indicates the physical duration of the first interval of 500 ms. Error bars are SEM.

We next aimed to further corroborate the functional role of temporal compression by testing object correspondence when visual-tactile stimuli are close to each other. A temporal interval was defined again by a tactile and a visual marker. The tactile interval marker consisted of a simultaneous impulse to the thumb and the index finger (see Fig. 3A). The visual interval end marker consisted of two dots flashed simultaneously, either in the same locations as the tactile stimuli (see Fig. 3A) or such that they formed an angle, which was orthogonal to the angle of the tactile stimuli (see Fig. 3A). If the functional role of temporal compression is to bind corresponding objects then compression should occur when the multisensory stimuli have the same orientation.

Figure 3C shows the perceived durations of the probe interval for the two orientations of the visual stimuli. The black bar illustrates perceived interval durations when visual and tactile stimuli were oriented in the same way while the gray bar shows perceived interval durations for visual and tactile stimuli oriented differently. When visual stimuli were presented at the same positions as the tactile stimuli, the perceived interval duration was shorter as when visual and tactile stimuli were presented at different positions. In the “same” condition, the perceived duration of the probe interval was underestimated in relation to its physical duration, while in the “different” condition judgments of the interval duration were almost veridical. A paired t-Test of the perceived first interval duration revealed that the means of the two groups (same vs. different) differed significantly (t(8) = 2.11, p = 0.034).

### Experiment 3: Attention

The aim of Experiment 3 was to unravel why the mask induces temporal interval compression. We tested the idea that the mask might withdraw visual attention from the stimulus marking the interval’s end. We presented one stimulus in the left and one in the right part of the screen as an interval start marker. After 500 ms an interval end marker appeared. In 80% of all trials, the interval end marker was shown in the right part and in 20% of all trials in the left part of the screen. With this manipulation we aimed to direct subject’s spatial attention to the right part of the screen where the stimulus occurred in the majority of the trials. This manipulation was tested in trials with and without masking the interval end marker.

**Figure 4:**
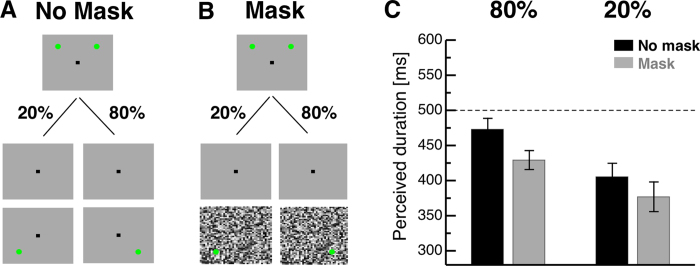
(**A**) Illustration of the first interval in the attention experiment. Two bars were presented to mark the interval start. After 500 ms the interval end marking stimulus was shown. This stimulus was presented in 20% of all trials in the left part of the screen and in 80% in the right part of the screen. (**B**) In half of the trials the stimulus marking the interval’s end was presented on top of a whole field mask. (**C**) Results from the attention experiment. Data are shown for the “80%” and the “20%” condition. Data from trials where the marker of the interval’s end was not masked are shown in black and data from masking trials in gray. Error bars represent SEM.

Average data from Experiment 3 are shown in Fig. 4C. In the “no mask – condition”, when the bar marking the interval’s end was presented in 80% of all trials in the right part of the screen, subjects estimated the interval duration nearly veridical (472.93 ms + −15.65 ms). In contrast, when the bar marking the interval’s end was presented in the left part of the screen in the remaining 20% of all trials, duration estimation was reduced to 407.20 ms + −14.24 ms. In the “mask condition”, interval duration was underestimated in the 80% condition (427.39 ms + −18.87 ms) and in the 20% condition (376.91 ms + −21.14 ms). A two-way repeated measures ANOVA of the perceived duration of the first interval was calculated with the factors “80%/20%” and “no mask/mask”. The spatial attention modulation changed temporal interval estimation as revealed by the significant factor “80%/20%” (F(1,14) = 8.31, p = 0.01). The significant factor masking (F(1,14) = 5.57, p = 0.03) confirmed that in the 80% condition where the occurrence of the interval end marker was expected, the mask produced time compression. No significant interaction was found, indicating additive effects of the 80%/20% - manipulation and the mask.

## Discussion

The main finding of this study is that multisensory temporal interval compression is selective for the correspondence of the interval markers. The estimation of temporal duration was strongly modulated by the spatial congruency of the interval markers. When two visual interval markers (dots) were flashed closely together in space and the last dot was masked the interval duration was underestimated by approximately 29%. Importantly, however, compression magnitude reduced as a function of spatial disparity between the interval markers. A very similar finding was observed when temporal compression was tested using visual and tactile stimuli to mark the interval. Interval duration was compressed by approximately 26% when the visual and the tactile stimulus were perceived to originate from the same position. In contrast, duration perception was close to veridical for large spatial separations between the visual and the tactile stimuli. In the multisensory setup, compression magnitude also depended on the feature correspondence between tactile and visual stimulation. When visual stimuli were close in space but non-correspondent to the tactile stimuli, the interval formed by these two stimuli appeared less compressed than when they corresponded.

**Figure 5:**
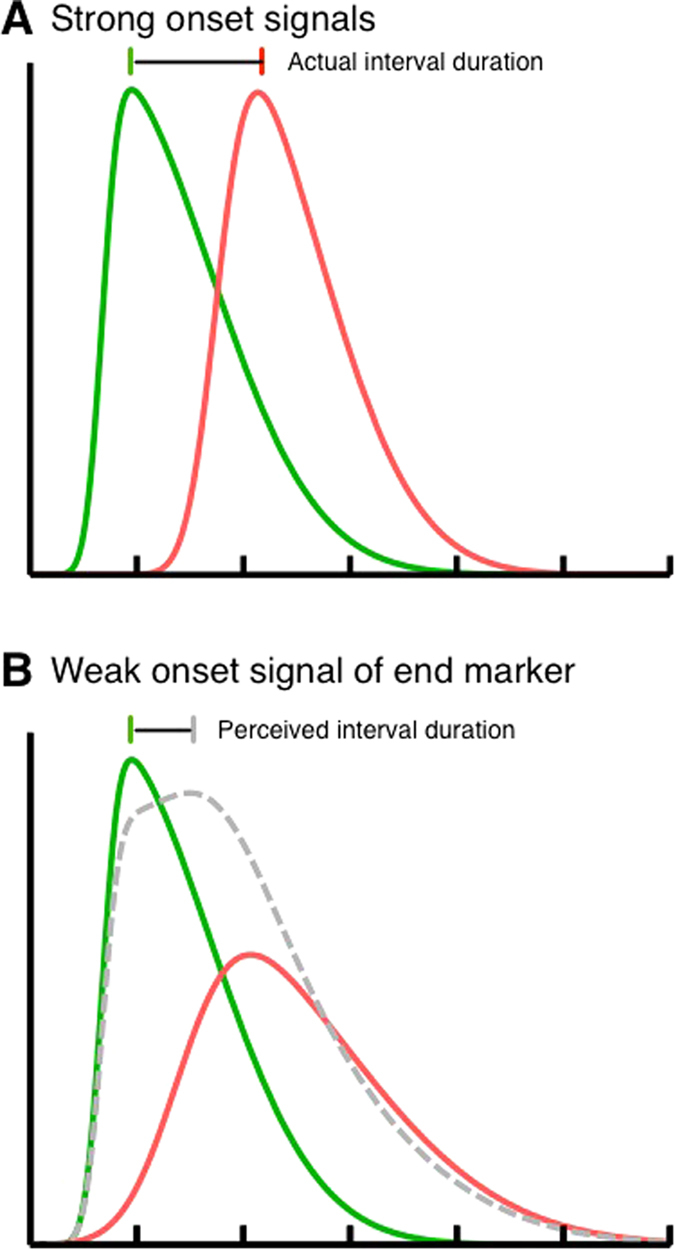
(**A**) Graphical illustration of the neural distributions responding to the occurrences of the interval start (shown in green) and the interval end (shown in red) marker. The panel shows distributions for strong onsets of both markers when no mask is presented. (**B**) Masking the interval end marker weakens its onset signal and the corresponding neural distribution becomes more variable. If the neural distributions responding to the strong onset signal of the interval start marker and the weak signal of the interval end marker are summed, the peak corresponding to the interval end marker shifts in direction of the interval start marker (shown in grey).

What might be the neural basis of masked-induced time compression? In order to estimate the interval duration, the occurrences of the interval markers must be compared to neural activity which somehow codes the elapsed time. The most prominent account of neural time representation is the ticking clock model^16^,^17^ (for an alternative model, see Mauk and Buonomano^18^. In this view the time point of objects or events is stored with reference to a tick of a neural clock and the duration of temporal intervals can be estimated by counting the number of ticks that occurred between interval start and end marker. If the interval markers have a strong onset transient signal, precise links between the occurrences of the marker and the tick can be established (see Fig. 5A). Weak onset signals however introduce variability in the linking procedure (see Fig. 5B, red curve). In our probe interval, the interval end marker was presented on top of a whole field mask which strongly impoverished the end marker’s onset strength and thereby made the stimulus harder to localize in time. We argue that variability in the neural distribution responding to the stimulus onset may be minimized by attraction towards a reference stimulus with a sharp peak and a narrow spread. On a behavioral level this attraction manifests as a shift of probe stimuli in space and time. On the neural level the attraction might be realized by a summation of the broad response distribution of the masked stimulus and the narrow response distribution of the stimulus that was not masked (see Fig. 5C, grey curve). Compression occurs if the interval markers have corresponding features. The integration of the interval markers might take place in multisensory receptive fields which are selective for the features of the markers. The functional benefit of this selectivity is a temporal sorting of corresponding objects.

These results are reminiscent of the literature on temporal order judgments in visual-tactile ventriloquism^14^. Subjects were more accurate to judge whether a visual or a tactile stimulus came first, when these stimuli were spatially separated rather than when they came from the same location. This result has been explained by a binding of visual and tactile stimuli into a unitary multisensory percept. We have chosen a comparably long interval duration of 500 ms, which is outside the binding range of ventriloquism effects. However, our compression effect did not produce a unitary multisensory effect, even when shorter probe intervals were tested (Zimmermann *et al*.^15^). We therefore argue that ventriloquism and temporal compression are separate phenomena. The difference might also be related to differential task demands. In our temporal compression task we instructed subjects to draw attention to the interval duration while in ventriloquism experiments temporal order judgements are required. Task demands might also explain why Keetels and Vroomen^19^ did not find an effect of spatial disparity in visual-tactile temporal ventriloquism. They tested the influence of a distractor stimulus on perceptual performance. Attention to the multisensory stimuli was not demanded. Neither was space relevant for the task. By contrast, our setup required participants to attend to the stimuli from both modalities in order to estimate the interval duration. Our results suggest that temporal compression rather than simply being a by-product of reduced allocation of temporal resources is likely to underlie the observed effects. We recently showed that temporal compression was significantly weakened when the interval marking stimuli were made dissimilar^15^. Here we extend these findings to multisensory integration. Data suggest that for short time intervals the correspondence between the visual and the tactile information as belonging to the same object/event governs their perceived location in time. If visual and tactile stimulation are spatially congruent and therefore enter the same multisensory receptive field, they are referred to as belonging to the same object/event. Several brain regions contain bimodal neurons with receptive fields responding to visual as well as tactile stimuli. Those neurons are probable candidates for multi-sensory integration processes as observed here. Bimodal neurons were found in ventral premotor cortex^20^, in parietal region 7b^21^,^22^, in the ventral intraparietal region^23^, and in the superior temporal region^24^. Consistent with these findings, fMRi studies revealed equivalent bi-modal activations in the posterior parietal cortex^25^,^26^.

The data from our Experiment 3 showed that temporal compression occurred when attention was drawn away from the interval end marking stimulus. The mask, acting as a salient distractor, might similarly have reduced attention toward the interval end marking stimulus. However, this interpretation has to be treated cautiously since the absence of an interaction effect in Experiment 3 suggests additive effects of the spatial attention manipulation and the masking. In this view, spatial attention drawn away from the interval end marking stimulus led to a compression of the interval duration. This account is consistent with studies in which spatial attention shifts away from the temporal interval stimulus led to duration underestimation^3^,^4^,^5^. These results may seem to be inconsistent with the predictability account, according to which unpredictable stimuli appear to last longer^8^, since in our Experiment 3 the unpredictable interval was compressed rather than dilated. However, first, in our experiments it was always the interval end marking stimulus and not the whole interval that was unpredictable. Second, it was the spatial and not the temporal position of the marker that was unpredictable. Another factor that might influence the perceived timing of the interval marker is the masking of its onset transient^27^,^28^.

In cases of visual disruptions, like eye movements or shifts of attention, the problem of object continuity occurs. As experiments on change detection^29^ have shown, only the spatiotemporal properties of objects that are attended to are registered consciously. We suggest that for the temporal features outside of the attention focus, temporal binding of corresponding objects provides object continuity and thereby establishes which objects belong together in the visual scene.

## Materials and Methods

### Apparatus

Figure 1A shows the experimental set-up. Stimuli were presented on a 23 inch HannsG HS233H3B LCD monitor. The screen resolution was set to 800 × 600 pixels with a refresh rate of 60 Hz. The screen was fixated upside down in a plexiglas construction with an angle of 45° and stimuli were viewed via a mirror that concealed the subject´s hand. This mirror was fixated 34 cm below and in parallel to the screen. Another 10 cm below and in parallel to the screen was a plane on which the subjects could place their hands. Positions of tactile stimulation of the hand matched the positions of the visual stimuli as viewed in the mirror. Subjects were disallowed to see their hands. The viewing distance to the mirror was 45 cm and the reflection of the screen in the mirror was 28 × 16 cm, resulting in a visual field of 35 × 20°. A Tactile Controller 2011TB by Heijo Research Electronics effected tactile stimulation. Small magnetic metal cylinders with a diameter of 0.4 cm were fired against the subject´s fingertip out of a larger cylinder with a diameter of 1.5 cm. Subjects wore headphones during the entire experiment to ensure that potential noise from the tactile stimulation did not affect performance.

### Experiment 1: Visual-tactile temporal compression

#### Participants

Seven subjects (6 female, one male, including two authors (EZ, CD), mean age 25 years) participated in the condition where the right hand was tested and six subjects (5 female, one male, including two authors (EZ, CD), mean age 25 years) in the condition where the left hand was tested. All had normal or corrected to normal vision and all except the authors were naive to the purpose of the experiment. Written informed consent was obtained prior to each experiment in accordance with the Declaration of Helsinki. Participants were remunerated for their time. All experiments of the study was approved by the ethics committee of the German Society of Psychology (DGPS) and conducted in accordance with their guideline.

#### Procedure

Stimuli were presented against a grey background. A fixation point (0.86 × 0.86°), illustrated by the square in Fig. 1B,C was first placed 9° to the left of the center of the screen. Subjects had to maintain fixation at this point. After a fixation period of one second, a tactile probe stimulus was delivered with a duration of 50 ms to either the left (Fig. 1B) or the right (Fig. 1C) index finger. The tactile stimulus was followed by an interval of 500 ms (Fig. 1D). The end of the interval was marked by a visual stimulus (1.3° diameter) at the center of the screen presented for 16 ms. This visual stimulus was covered immediately by a mask with 50 ms duration. After 1000 ms, another tactile stimulus appeared at the same position as the first one, followed by an interval with a variable duration ranging from 200 to 800 ms in steps of 50 ms that once more was terminated by a visual stimulus. Subjects were instructed to judge which of the two time intervals was shorter by using the computer keyboard (two alternative forced choice task). The perceived duration of the first interval was measured with an adaptive staircase method (Best PEST).

#### Stimulus positions

If bimodal neurons are involved in the putative effect of visual-tactile temporal compression then the spatial principle of multisensory integration should govern the effect. This principle states that multisensory neurons are activated only if stimulation enters the separate modalities from the same region in external space, since their receptive fields are in spatial register^25^. To this end, we varied the spatial position of the tactile stimulation by placing the subject’s hand blockwise in separate locations: In blocks where the fixation point was presented on the left side of the screen, the hand was placed either 0°, 12°, or 24° to the right of the screen center. When the fixation point was presented on the right side of the screen, the hand was placed either 0°, −12°, or −24° to the left of the screen center.

In total, we manipulated three factors: The hand to which we presented the tactile stimulus (left vs. right hand), the side of fixation (left vs. right side), and the three positions at which we presented tactile stimuli (0° vs.12° vs. 24°). This resulted in a total of 12 experimental conditions, which were tested in blocks of 40 trials each with a duration of 3 minutes. We divided subjects into two groups by side of fixation. The factors hand and position of tactile stimuli were tested as within-subjects factors. The presentation order of the experimental conditions was counterbalanced between subjects and each subject did one preparation block that was also counterbalanced and not used for further calculations. For our analyses, we averaged over the last 10 trials of each block to obtain a reliable threshold value.

### Experiment 2: Correspondence matching

#### Participants

Nine subjects (three males and six females, mean age 25 years, including two authors (EZ, CD)) with normal vision participated in the experiment. All participants except for the authors were naive to the purpose of the experiment.

#### Procedure

The trial setting was the same one as in Experiment 1. The two intervals were now initiated by two tactile stimuli, presented simultaneously for 50 ms. Intervals were ended by two visual stimuli, presented simultaneously for 16 ms. Tactile stimuli were always presented at the same positions: one 2.16° right and 2.16° above the center of the screen, the other 2.16° left and 2.16° below the center of the screen. Visual stimuli were presented either at the same positions as the tactile stimuli (same condition) or on the opposite side at an equivalent eccentricity as the tactile stimuli (different condition). In the different condition, visual stimuli were presented 2.16° right and 2.16° below the center of the screen, and 2.16° left and 2.16° above the center of the screen, respectively. The positions of the visual and tactile stimuli are shown in Fig. 3A,B. The first interval was fixed to 500 ms. The second interval varied between 200 to 800 ms, in steps of 50 ms. Visual stimuli at the end of the first interval were masked. The second interval followed the first interval after a break of 1000 ms. Like in the time condition, subjects indicated which interval appeared to be shorter using the computer keyboard. The perceived duration of the first interval was measured with the adaptive staircase method Best Pest (Lieberman & Pentland, 1982). Subjects completed two experimental blocks. In one block, tactile and visual stimuli were presented at different positions, and in the other block at the same positions. Each block consisted of 40 trials and had a duration of 3 minutes.

### Experiment 3: Attention

#### Participants

Fifteen subjects (7 males and 8 females, mean age 27 years, including two authors (EZ, CD) with normal vision participated in the experiment. All participants except for the authors were naive to the purpose of the experiment.

#### Procedure

Participants were required to keep their gaze on a fixation point (0.88°x0.88°) which was shown at the screen center. After 1000 ms two dots (radius: 1.36°) were presented, one in the left and one in the right part of the screen at a horizontal eccentricity of 9° and 6.8° above the horizontal meridian. The dots marked the start of the probe interval and were shown for 16 ms. After 500 ms a single dot (radius: 1.36°) was shown, which marked the end of the interval. In 80% of all trials this dot was shown in the right part of the screen and in 20% of all trials in the left part of the screen at the same horizontal eccentricity as the first two dots and 6.8° below the horizontal meridian. In half of all trials, the bar marking the interval end was masked by a visual whole field mask, which was presented for 50 ms (Fig. 4A,B). After 900 ms a second interval was presented, marked by identical stimuli as in the first interval. No mask was presented after the second interval. The second interval had a variable duration ranging from 250 to 750 ms in steps of 25 ms. Spatial positions of the dots marking the beginning and the end of the second interval were identical to the first interval and therefore 100% predictable. Participants had to judge which of the intervals was shorter by using the computer keyboard (two alternative forced choice task). The perceived duration of the first interval was measured with a Best PEST staircase method.

### Ethical statement

Written informed consent was obtained prior to each experiment in accordance with the Declaration of Helsinki. Participants were remunerated for their time. All experiments of the study was approved by the ethics committee of the German Society of Psychology (DGPS) and conducted in accordance with their guideline.

## Additional Information

**How to cite this article**: Zimmermann, E. *et al*. The functional role of time compression. *Sci. Rep.* **6**, 25843; doi: 10.1038/srep25843 (2016).
